# Young Women with Breast Cancer: The Current Role of Precision Oncology

**DOI:** 10.3390/jpm13111620

**Published:** 2023-11-20

**Authors:** Amirrtha Srikanthan, Arif Ali Awan, Sharon McGee, Moira Rushton

**Affiliations:** 1 Division of Medical Oncology, The Ottawa Hospital, 501 Smyth Road, Ottawa, ON K1H 8L6, Canada; aawan@ohri.ca (A.A.A.); shmcgee@toh.ca (S.M.); moirushton@toh.ca (M.R.); 2 Department of Medicine, Faculty of Medicine, University of Ottawa, 451 Smyth Road, Ottawa, ON K1H 8M5, Canada

**Keywords:** young adults, breast cancer, precision oncology

## Abstract

Young adults aged 40 years and younger with breast cancer represent less than 5% of all breast cancer cases, yet it is the leading cause of death among young women with cancer worldwide. Breast cancer that develops at a young age is more aggressive and has biological features that carry an increased risk of relapse and death. Young adults are more likely to have a genetic predisposition and key biomarkers, including endocrine receptors, the HER2 receptor, and proliferation biomarkers, that appear different compared to older adults. Despite being more aggressive, management strategies are largely the same irrespective of age. Given the higher rates of genetic predisposition, fast access to genetic counselling and testing is a necessity. In this review, the biological differences in young adult breast cancer and the current role precision medicine holds in the treatment of young adults with breast cancer are explored. Given the relatively high risk of relapse, developing novel genomic tools to refine the treatment options beyond the current standard is critical. Existing predictive genomic tests require careful interpretation with consideration of the patient’s clinical and pathological features in the young patient cohort. Careful evaluation is also required when considering extended endocrine therapy options. Improved characterization of mutations occurring in tumors using next-generation sequencing could identify important driver mutations that arise in young women. Applying the advances of precision medicine equitably to patients in resource-rich and low- and middle-income countries will be critical to impacting the survival of young adults with breast cancer worldwide.

## 1. Introduction

Breast cancer in the young adult (ages 18–39 years) population is rare, representing less than 5% of all cases, with the median age of a breast cancer diagnosis being in the early 60s [1]. However, breast cancer is the most frequent form of cancer affecting young women younger than 40 years of age throughout the world and one of the leading causes of cancer-related death [2]. In addition, breast cancer rates in younger women continue to rise despite stability of rates in older women [3]. Young age of onset is recognized as an independent factor for poor prognosis [4]. Tumors are often larger, of higher grade, and frequently present with regional or distant spread [5,6]. Young adults with breast cancer also exhibit differences in molecular subtype compared to older women and [6] are more likely to have aggressive tumors [7]. Furthermore, most breast cancer screening programs begin at the age of 50 years, with none at younger than age 40 years in the absence of hereditary syndromes [8]. Collectively, these factors contribute to breast cancer being the leading cause of cancer-related death in women younger than age 40 years, with survival rates among young adults with breast cancer lower than those of older women, even when given comparable treatments [9].

Precision medicine, the approach of customizing disease prevention and treatment by integrating the unique molecular or genomic differences in individuals, serves many roles in breast cancer management [10]. Through the routine identification of biomarkers that tailor treatment, to genomic testing that allows for prognostication [11,12], examples of precision medicine can be found in the management of adjuvant and metastatic breast cancer [6]. In breast cancer management, advances in precision medicine have already resulted in approved treatments that are tailored to the specific characteristics of a patient, such as a person’s genetic makeup or the genomic profile of their tumor. Despite progress in precision medicine and the recognition that young adults with breast cancer experience more aggressive tumors with poorer prognosis, further advances are needed to tailor treatment to young adult breast cancer disease biology and recurrence risk. In this review we will explore the biological differences in young adult breast cancer and the current role precision medicine holds in the treatment of young adults with breast cancer.

### Biology of Breast Cancer in Young Adults

Cumulative evidence demonstrates that young adults with breast cancer exhibit differences in breast cancer biology compared to older adults (Table 1). Large prospective observational studies have been undertaken assessing the pathological features identified in young women younger than 40 years of age at diagnosis [13]. The Prospective Study of Outcomes in Sporadic and Hereditary Breast Cancer (POSH) study assessed 2956 patients between 2000 to 2008. The median age of diagnosis was 36 years. Of these women, the majority had ductal histology (86.5%) and grade three tumors (58.9%). The median tumor size was 22 mm, half of the patients were node positive (50.2%), and multifocality was observed in 27%. Estrogen receptor (ER) status was negative in 33.7%, and 24% were human epidermal growth factor receptor 2 (HER2) positive. The Young Women’s Breast Cancer Study demonstrated similar results [14]. This study assessed 1297 women recruited from 2006 to 2016, with a new diagnosis of breast cancer at age 40 years or younger. The median age of diagnosis was 37 years. Most of the recruited patients were White (85%). Approximately 58% of invasive tumors were high grade. With respect to subtype, 32.9% were luminal A, 42.4% luminal B, 8.3% HER2-enriched, and 16.4% triple-negative. There were no differences in molecular phenotype, stage, grade or histopathology between the different age groups (≤30 years, 31–35 years, and 36–40 years). Germline BRCA mutations were found in 11% of tumors, of which 64.1% were BRCA1 carriers (63.1% triple-negative) and 35.9% were BRCA2 carrier (55.3% luminal B). The Canadian Reducing the Burden of Breast Cancer in Young Women (RUBY) study is an ongoing prospective study that aims to learn more about the impacts of biology, genetics, lifestyle, and treatment on outcomes in this population [15].

Several retrospective studies have also evaluated differences in pathological features based on age. A large analysis was undertaken of more than 200,000 patients, of whom approximately 15,000 were younger than age 40 years at diagnosis, from the US Surveillance, Epidemiology, and End Results database [16]. This study identified that compared to older adults (≥40 years), young adults more commonly were diagnosed with tumors that were: large (*p* < 0.0001), poorly differentiated (*p* < 0.0001), lymph node positive (*p* < 0.0001), and ER negative (*p* < 0.0001). Additionally, a population-based study from the California Cancer Registry, which included 5,605 patients aged younger than 40 years at diagnosis, showed higher HER2 expression in younger patients [17]. Multiple studies in hospital-based settings internationally corroborate these findings [18,19,20] in that breast cancer diagnosed in young adults has more aggressive pathological features.

Breast cancer is recognized as a heterogenous disease with at least four intrinsic subtypes: luminal-A, luminal-B, basal-like and HER2-enriched subtypes [21]. Gene expression profiling has been undertaken to characterize the pattern of breast cancer molecular subtypes in young adults with breast cancer. Young adults had a significantly higher proportion of higher risk basal-like tumors: 34.3% in those younger than 40 years compared to 27.7%, 20.8%, and 17.9% in the other age groups (41 to 52, 53 to 64, and ≥65 years, respectively) (*p* < 0.0001). A higher proportion of HER2-enriched tumors was also identified in young patients. Conversely, young adults were less likely to have more favorable risk luminal-A tumors compared to older age groups: 17.2% compared to 30.7%, 35.1%, and 35.4% (41 to 52, 53 to 64, and ≥65 years, respectively) (*p* < 0.0001) [7].

Studies have also used immunohistochemical surrogates with varying definitions to assess tumor subtypes in young adults with breast cancer. The distribution of subtypes observed varied between these hospital-based studies. For example, basal-like tumors ranged from 19 to 38% [6]. Collectively, these studies demonstrate that there is a lower prevalence of ER-positive/HER2-negative tumors in younger patients but a high proportion of triple-negative tumors, and HER2 over-expression irrespective of ER status.

Complicating the understanding of biological differences across the breast cancer spectrum are the definitions used in studies. ‘Young age’ has often been synonymous with ‘premenopausal’ which can include women younger than age 50 years. Evaluations have been undertaken in the broader ‘premenopausal’ age range. In a large analysis of 1427 patients, aggressive features were more frequently identified in tumors of premenopausal patients [18]. Similar findings were identified in a large study from the Korean Breast Cancer Society registry including 9885 patients 50 years or younger at diagnosis [22]. When assessing young women 40 years or younger, no significant differences were identified in histological features or ER, progesterone receptor (PR), and HER2 expression between patients 30 years or younger, 31 to 35 years and 36 to 40 years [23]. Collectively, these findings suggest that there are more aggressive tumor features in young adults with breast cancer; however, the distinctions appear to occur below the age of 35 or 40.

## 2. Molecular Profiling in Young Adults with Breast Cancer

Studies utilizing gene expression profile comparison have identified specific genes and molecular profiles that could help identify unique factors associated with younger women with breast cancer. One of the first attempts looked at 200 patients aged 45 years or younger compared to 211 patients aged 65 years and older. These studies initially suggested that there are differences in expression patterns between tumors in younger versus older women. However, when these older studies were reanalyzed, the conclusions were questioned [24]. A higher probability of PI3K (*p* = 0.006) and Myc (*p* = 0.03) pathway deregulation was identified in tumors arising in younger patients when originally evaluated. However, the original analysis was not adjusted for known prognostic factors, such as differences in intrinsic subtypes. A similar analysis was repeated by the same group with adjustment for intrinsic subtypes. This repeat analysis identified that younger patients had more basal-like tumors. However, after adjustment for subtype differences, no distinct age-related molecular differences could be identified.

A more recent pooled gene expression analysis evaluating two datasets including 1188 (≤40 years  =  191) and 2334 (≤40 years  =  260) patients was published in 2012 [7]. This work assessed the association between age and gene alterations identified through literature searches to be related to early-onset breast cancer (breast cancer presenting prior to the age of 45) [25]. The analysis was adjusted for differences in intrinsic subtype, histological grade, tumor size, and nodal status. These independent datasets demonstrated that young patients had higher expression of c-kit (*p* < 0.001), RANK-ligand (*p* < 0.0001), mammary stem cell progenitors (*p* < 0.0001), and luminal progenitors and germline(g) BRCA1 mutation signatures (*p* = 0.007). Increased disruption of the MAP kinase and PI3K pathways (*p* < 0.0001) was identified, in addition to lower expression of gBRCA1 (*p* = 0.003) and apoptosis-related genes, particularly FAS (*p* = 0.03). These alterations in growth pathways and DNA repair may provide some explanation for the more aggressive breast cancer phenotype seen in young women.

The differences identified may be explained by the more contemporary datasets including four times more patients. Additionally, the prior paper utilized an unbiased approach in searching for genes associated with age. This approach requires higher numbers of patients, due to adjustment for confounders and multiple comparisons [24]. The results from the recent datasets suggest insights into the biology of young adults with breast cancer [7]. For example, high gBRCA1 mutation signature expression is consistent with the higher prevalence of gBRCA1 mutations in young patients [26,27]. Patients with BRCA1 mutations are also more commonly diagnosed with basal-like tumors [28]. Earlier research has suggested that luminal progenitors may be the cell of origin in these tumors [29]. The higher expression of gBRCA1 mutation signatures and luminal progenitors in young patients may explain why young adults develop basal-like tumors at higher frequencies.

Several studies have made attempts to understand the somatic mutations of breast cancer. Next-generation sequencing (NGS) has identified point mutations in TP53 and PIK3CA genes, which account for 25% of cases [30,31]. Less is known about the mutation patterns of young women. Whole-genome sequencing of 100 breast tumors found no correlation between age at diagnosis and total somatic base substitutions for ER-positive and ER-negative tumors [32].

Ongoing studies have focused on specific genes that have been linked to aggressive breast cancer at a young age, such as gBRCA1 and TP53. There is also investigation into the role of the tumor microenvironment/stroma in the initiation and progression of breast cancer in young adults [33]. Studies of molecular mechanisms of breast cancer subgroups in all adults can be informative for young adults as well, as no consistently unique young adult factors have been definitively identified [34]. Carefully controlled analysis of gene expression signatures in young adult tumors relative to the same subtypes in older patients are needed to determine if a specific signature is linked to young adult breast cancers [35]. Additionally, further studies evaluating potential biologic differences based on ethnic background in the young adult population are needed. It is recognized that certain ethnicities, such as African Americans, are at increased risk of aggressive triple-negative breast cancer [36,37]. Research to understand the biology and genetics of breast cancer as it relates to ethnic identity are important. Detailed studies of triple-negative/basal subgroups are particularly important as these breast cancers are more frequent in young adults [38]. Whole-genome analysis with deep sequencing may also identify mutations or polymorphic patterns that could be linked to breast cancer susceptibility in young adults [39].

## 3. Impact of Precision Medicine on the Clinical Management of Young Adults Affected by Breast Cancer

### 3.1. Genetic Predisposition

Young adults with breast cancer are more likely to have underlying genetic conditions that contribute to the higher chances of developing malignancies [40]. Cancer predisposition syndromes such as Li–Fraumeni and germline mutations in inherited breast and/or ovarian cancer genes commonly lead to the development of cancer among young adults. Approximately half of young adults with breast cancer diagnosed before 30 years have germline BRCA1, BRCA2, or TP53 mutations [41]. These mutations are associated with up to a 70% lifetime risk of breast cancer [42]. In a large sample of over 21,000 families who met German BRCA1/2 mutation testing criteria, a germline mutation was identified in 13.7% of families who presented with a single case of breast cancer diagnosed at younger than 36 years [43]. The emphasis on initiating screening earlier among individuals with hereditary syndromes compared to standard population recommendations reflect the early age of onset observed in hereditary breast cancer [44,45].

The identification of underlying genetic conditions has a direct impact on clinical care. Individuals with hereditary syndromes are at increased risk of early-onset in addition to bilateral breast cancer. The pathogenic variants associated with these hereditary syndromes are considered highly penetrant [41].

Local therapy decisions (i.e., surgery) are influenced by the presence of a known mutation. Thus, it is critical that genetic counselling and testing be offered to young adults as soon as possible after a breast cancer diagnosis. Guidelines recommend considering risk-reducing bilateral mastectomy among patients with BRCA1/2, PALB2, TP53, and other germline mutations predisposing to breast cancer [41]. In addition, prophylactic bilateral salpingo-oopherectomy is recommended between the ages of 35 to 40 years and at age 40 years in BRCA1 and BRCA2 carriers, respectively, after the completion of childbearing [41].

Information about genetic alterations also informs systemic therapy options with PARP inhibitors, approved for use in gBRCA1/2 mutations—which are more likely to be harbored by young adults. In the OLYMPIA trial, which randomized 1836 patients with high-risk HER2-negative early-stage breast cancer and gBRCA1/2 mutations to 1 year of adjuvant olaparib or a placebo, olaparib significantly improved invasive disease-free survival (3-year rate, 85.9% vs. 77.1%; *p* < 0.001) and overall survival (4-year rate, 89.8% vs. 86.4%; *p* = 0.009) [46,47]. PARP inhibitors have also demonstrated benefits in the unresectable advanced/metastatic breast cancer setting, for patients with genetic mutations [48,49]. The use of precision medicine in genetic testing for young adults with breast cancer has led to tailored clinical management strategies in prevention and treatment that have directly improved mortality.

### 3.2. Integrating Precision Medicine through Genomic Testing

Multiple genomic tests are available to improve prognostication and guide decision-making around systemic therapy in the adjuvant setting for breast cancer [21]. Several studies have investigated the prognostic performance of the Oncotype DX, Breast Cancer Index (BCI), Prosigna, MammaPrint, and EndoPredict genomic assays in various settings [50,51]. This includes the immediate adjuvant setting as well as the late recurrence setting (>5 years after diagnosis) [50].

These tests add further information to classic clinicopathologic prognostic variables in patients with ER-positive tumors and reliably distinguish between patients at low and high risk of recurrence [51]. Given their reliability and cost-effectiveness [52], they are considered standard clinical practice to differentiate those who will derive benefit from chemotherapy and those who will not and can therefore safely avoid the toxicity from chemotherapy.

Despite their utility, concerns about whether these assays have the same prognostic value in young adults have been raised, as these signatures were mainly developed using the postmenopausal female population. The initial work on MammaPrint, for example, included 295 patients, of whom only 63 (21%) were younger than 40 years. Of these 63 patients, 52 young adult patients (82%) were classified as high risk [53]. The same finding was observed in earlier studies with Oncotype DX, where only 59 out of 668 (8.8%) patients were younger than 40 years, yet the majority of young patients had a high-risk score (33/59; 56%) [11]. This finding was higher than the proportion of high-risk scores in patients aged 40 to 50 (29%), 50 to 60 (25%) and >60 years (21%). Other genomic signatures were also developed using populations of older patients; thus, extrapolating from these studies and determining the value of these signatures in the young population is challenging. Due to the minority of patients aged 40 years and younger represented in validation studies, the adoption of genomic testing in young adults with breast cancer has lagged behind older women [54].

In premenopausal patients, clinicians may use Oncotype DX in patients with node-negative ER-positive and HER2-negative breast cancer. Those with low recurrence scores (RS) can forego chemotherapy. However, in the TAILORx trial, the addition of chemotherapy to endocrine therapy was associated with a decrease in the 9-year rate of distant recurrence in node-negative patients age 50 years or younger with RS 21–25 (difference, 6.4 ± 4.9%) and RS 16–20 if the clinical risk was high (difference, 6.5 ± 4.9%) [55,56]. This contrasts the main analysis, which demonstrated endocrine therapy alone was effective for tumors with intermediate RS. Additionally, there was no difference observed in distant relapse-free survival at 6 years by receipt of chemotherapy among node-negative patients age 40 and younger with RS 11–25 enrolled in the Young Women’s Breast Cancer Study, a prospective observational cohort [57]. Patients with and without chemotherapy in this setting had a good prognosis. It is challenging to determine whether patients treated with chemotherapy in this retrospective group may have had other higher risk features that resulted in benefit from chemotherapy.

In RxPONDER trial, chemotherapy in addition to endocrine therapy in premenopausal women with one to three involved nodes and RS 0–25 was associated with an improved 5-year rate of invasive disease-free survival (93.9% vs. 89.0%; *p* = 0.002) and distant relapse-free survival (96.1% vs. 92.8%; *p* = 0.0009). However, postmenopausal women with these same characteristics could safely forego chemotherapy [58]. A similar finding was identified using the MammaPrint genomic test. Women aged 50 years and younger with high clinical risk and low genomic risk had improved distant metastasis-free survival at 8 years with the addition of chemotherapy to endocrine therapy versus endocrine therapy alone in the recently updated MINDACT trial. No chemotherapy benefit was seen in the over-50 age group [12].

Of relevance, only 13–15% of premenopausal women who received endocrine therapy on these trials received concurrent ovarian function suppression. Thus, questions remain regarding whether the survival advantage young adults derived from chemotherapy was a result of ovarian suppression as opposed to direct cytotoxicity. Chemotherapy-related amenorrhea is a well-established predictor for improved disease-free survival and overall survival in premenopausal patients with hormone receptor-positive disease [59,60]. The higher incidence of chemotherapy-related amenorrhea in older premenopausal adults may explain the beneficial impact of chemotherapy in patients ages 41–45 and 46–50 years with an intermediate RS but not in those age 40 years and younger in a subgroup analysis of the TAILORx trial [56,59]. The currently accruing OFSET study is attempting to answer this question. In this study, all premenopausal women with ER-positive, HER2-negative early-stage breast cancer receive an aromatase inhibitor with ovarian function suppression. Those with an RS of 25 or less (if they have positive lymph nodes) or 16–25 (if they are lymph node negative) are randomized to receive or omit chemotherapy [61]. 

Until more data are available to guide the optimal use of chemotherapy in young women with breast cancer, some experts recommend an individualized approach for intermediate-risk patients. This approach would consider clinicopathologic risk factors and the risks and benefits of adjuvant chemotherapy, particularly when maximal endocrine therapy is planned. One such strategy includes using neoadjuvant endocrine responsiveness, determined by change in Ki67 score to short course pre-operative endocrine therapy, coupled with the Oncotype DX RS. Using such an approach enables sparing of chemotherapy usage in pre- and post-menopausal women with ≤3 affected lymph nodes [62].

After 5 years of adjuvant endocrine therapy, some patients with hormone receptor-positive breast cancer still have a significant risk for late recurrence [63], including distant metastases, that might be prevented with longer durations of endocrine therapy [64]. However, the added toxicity and variable benefit derived from extended endocrine therapy make optimal patient selection crucial. Genomic assays are in development to risk-stratify patients for late recurrence and determine the efficacy of extended endocrine therapy [65], with the aim to help guide extended endocrine therapy decisions for clinicians and individualize treatment strategies for patients. Furthermore, young women are less likely to be adherent to endocrine therapy than older women due to increased treatment-related toxicities including vasomotor symptoms. Thus, accurate identification of patients who will benefit from extended therapy can aid with adherence counselling [66]. Strategies are needed to clearly identify those who will benefit from ovarian suppression and extended adjuvant therapy. While some tools already exist (e.g., CTS-5 score), integrating molecular and genomic information may be of value and is an area of future research [67,68].

### 3.3. Increasing Role of Precision Medicine for Young Adults with Metastatic Breast Cancer

In the metastatic setting, the standard of care integrates biomarker testing with treatment regimens determined by ER and HER2 status. By targeting driver alterations, we have achieved significant improvements in survival in advanced breast cancer. For example, systemic therapy for advanced hormone receptor-positive, HER2-negative breast cancer has dramatically changed over the past decade. CDK 4/6 inhibitors in combination with endocrine therapy (e.g., aromatase inhibitors +/− ovarian suppression) are now the standard of care in the first line setting for these patients, with data showing definitive survival benefits in young pre- and peri-menopausal females with this approach [69,70]. Determining the optimal sequence of systemic therapy after progression on a first-line therapy for hormone receptor-positive, HER2-negative breast cancer is an evolving space.

NGS technologies have evolved such that the mutational landscape of tumors can be profiled with relatively reasonable costs and time frames. This increased accessibility enables the integration of NGS into clinical care delivery [71]. In metastatic breast cancer, identifying actionable genomic alterations can lead to the tailored use of effective new therapies in a rational and sequential manner, thus prolonging survival and delaying the requirement for more toxic chemotherapy [72].

The sequence of treatment after progression on a previous CDK 4/6 inhibitor and endocrine therapy is becoming more complicated due to increasing therapeutic options. Recognizing this complexity, expert opinion papers in breast cancer provide guidelines to help clinicians prioritize treatment options [73,74]. This remains an active area of research, with numerous ongoing clinical trials investigating targeted therapies in the post CDK4/6 inhibitor space. Based on expert opinion, ERBB2 amplification, BRCA1/2 mutations, and PIK3CA mutations have evidence for targeted therapies supported by large randomized clinical trials. ESR1 mutations and PTEN loss have drug matches associated with antitumor activity; however, the magnitude of benefit is unknown [73]. Other potential driver mutations exist, although are less common (Table 2). If access to clinical trials and/or targeted agents such as PIK3CA inhibitors, AKT inhibitors, oral SERDs, and PARP inhibitors are available, NGS for genomic alterations should be made available to patients [72], thus allowing for precision medicine to tailor treatment. Generally, systemic treatment options after the development of endocrine-resistant disease are limited to the sequential use of single-agent chemotherapy.

In the metastatic triple-negative setting, integration of genetic testing, for example, allows for the use of PARP inhibitors in patients with gBRCA1/2 mutations [48,49]. PDL-1 status is also used as a marker to determine which individuals may or may not benefit from immunotherapy [75]. In the HER2-positive and HER2-low setting, various therapies targeting HER2 expression have demonstrated improvements in survival [76,77]. Although clear examples of precision medicine advancements are available for patients with metastatic breast cancer with all subtypes, the treatment approach remains similar across ages.

## 4. Ethical Considerations

In regions where populations are much younger and population-based screening is not routine compared to North America, such as Africa and the Middle East, up to 20% of patients are diagnosed with breast cancer below the age of 40 years [19,78,79]. It is unknown whether underlying genetic differences or environmental factors result in young women in Africa and the Middle East being more prone to breast cancer development and is thus the subject of ongoing research [80]. Furthermore, rates of breast cancer among young women are increasing globally, driven by countries with lower resources [2].

Much of the current understanding regarding breast cancer biology and management is driven by data from North American and European countries. There is increasing data from Asian high-income countries that the host biology may be clinically relevant. For example, higher prevalence of luminal-B subtypes has been identified in Asian studies [81]. Even among countries with access to equivalent resources, the inclusion of more ethnically diverse patients is necessary to understand the degree of interethnic heterogeneity.

This poses ethical challenges as advances in care through precision medicine are made. The technical and human infrastructure needed for the diagnosis, treatment, and monitoring of cancers is suboptimal in affluent countries [71]; however, it is especially challenging in low- and middle-income countries [82]. NGS has rarely been applied to tumors from low- and middle-income countries, however, this remains an area of opportunity for the future. The capacity to detect cancer-associated mutations in the peripheral blood, through liquid biopsy for example, is a minimally invasive diagnostic tool using cell-free DNA and circulating tumor DNA (ctDNA). Emerging work has demonstrated the ability of ctDNA to monitor the tumor burden in women undergoing therapy for metastatic breast cancer [83]. Developing alternative diagnostic and treatment monitoring methods can be attractive in low- and middle-income countries given the limited availability of clinical pathology and radiography in these countries [82].

Even in high-income countries, there is a significant difference in outcomes for young women of racial/ethnic minorities diagnosed with breast cancer [84]. While many factors likely influence this outcome, this disparity highlights the need for more targeted research and clinical care for these populations to improve survival. Existing genomic testing tools have demonstrated lower prognostic accuracy in African American females in the US with Oncotype DX [85] or differing prognostic information between existing tools (Oncotype DX and MammaPrint) [86]. Better model calibration is required in racially and ethnically diverse populations to improve survival. As precision medicine continues to evolve and further advances are made in the management of young women with breast cancer, strategies to ensure these advances are valid across diverse populations and can be accessed globally are important.

## 5. Future Directions

Breast cancer that develops at a young age is more aggressive and has potentially distinct features that impact not only breast cancer risk but also breast cancer phenotype and biology. Despite this clear uniqueness, management strategies are often the same regardless of age. There is a need to develop a biology-driven approach to refine treatment for young adults with breast cancer [87]. Given the relatively high risk of relapse, developing novel genomic tools to tailor the treatment–decision process is critical. These tools can guide not only when to withhold chemotherapy when there is little benefit, but also identify who might be appropriate for extended adjuvant therapy. As up to 40 to 50% of young ER-positive patients relapse after 5 years [13], such tools can identify individuals who could derive greater benefit from extended adjuvant therapy. Finally, improved characterization of somatic mutations that occur in tumors arising in young adults using NGS may identify key driver mutations to target. Applying the advances of precision medicine equitably to patients in resource-rich and low- and middle-income countries will be critical to improving the outcomes of young adults with breast cancer worldwide.

## Figures and Tables

**Table 1:** Differences in the biology of breast cancer for young women.

Clinical-Pathological Features	Expression Profile	Genomic Profile
Higher grade Larger size Greater risk of lymph node involvement	Lesser likelihood to be luminal A Higher likelihood to be luminal B, basal, or HER2 enriched	Germline: higher likelihood of hereditary syndromes, particularly BRCA1/2 and Li–Fraumeni (TP53 mutation) Differential alterations in PI3K, MAPK, BRCA1/apoptosis, TP53, and RANK-L pathways

**Table 2:** Summary of currently actionable alterations in breast cancer [74].

Gene	Alteration	Targeted Therapy	Prevalence	Actionability
ERBB2/Her2	Amplifications	Her2-directed therapies	15–20%	Improved outcomes in clinical trials
PIK3CA	Hotspot mutations	PIK3CA alpha inhibitors	30–40%	
BRCA1/2	Germline	PARP inhibitors	4%	
Mismatch repair	MSI-H	Immune checkpoint inhibitors	<1%	
NTRK	Fusions	NTRK inhibitors	<1%	
ESR1	Hotspot mutations	Selective estrogen receptor degraders	10–20%	Anti-tumor activity but magnitude of benefit unknown
PTEN	Hotspot mutations	PIK3CA/AKT/mTOR inhibitors	7%	
High TMB	Hotspot mutations	Immune checkpoint inhibitors	3–5%	
ERBB2/Her2	Hotspot mutations	Small molecular inhibitors	2–4%	
AKT1	Mutation	AKT inhibitors	2–5%	
BRCA1/2	Somatic	PARP inhibitors	3%	Hypothetical benefit
